# The Principles of Medical Ethics

**Published:** 1903-07

**Authors:** 


### The Principles of Medical Ethics.

IT IS now a widely-known fact that the American Medical Association, at New Orleans last May, completely reversed the position to which it has clung so long in relation to the code of medical ethics. We made comment on this action at considerable length in the June issue of the JOURNAL, and need not further enter into a discussion on the subject now; but, inasmuch as the medical journals throughout the country are dealing with it in almost unanimous approbation, we invite the attention of our readers to the two views which are here reprinted,—one from the east, the other from the west,—and which, though differing in details, yet both reach the same general conclusions.

### THE AMERICAN MEDICAL ASSOCIATION AND THE CODE OF ETHICS.

[*Post-Graduate*, June, 1903.]

The American Medical Association has again spoken on the subject of ethics. Essentially the old code, as held by the New York State Medical Association, was presented by the committee appointed for that purpose, of which Dr. Ellsworth Eliot Harris was the chairman. This code was of the same general character as the one over which a controversy has been going on in the State of New York for a number of years, and yet more liberal as to consultations. Through the vigorous efforts of Dr. Reed, of Cincinnati, the name of Code was changed to the Principles of Ethics, and the subject of ethics and discipline was left to each State. Here the American Medical Association dodged an opportunity. The only proper course was to have made no mention of a code or principles of ethics. If the committee from the New York State Medical Association had been willing to do this, as everybody knows, the rest of the American Medical Association would have gladly accepted the proposal, but the seceding organisation of the State of New York could not allow its much treasured code to die so easily. After a long discussion, Dr. Reed's ideas were adopted, and the Principles of Ethics of the American Medical Association were made the report of the council and adopted unanimously by the association. The action of the Medical Society of the State of New York in finally declining, after three years discussion, in 1882, to make the old code a part of its by-laws, and finally in abolishing the written code, has been abundantly justified by this action.

The American Medical Association has virtually accepted the New York idea, and our society is vindicated from the unjust aspersions of the former body, which refused to receive our delegates after we had done less in the way of abrogating a code, than the association itself has now done. There is now nothing for the New York State Medical Association to do but to disband. It was organised to sustain the old code, but the old code died in 1882, and is now decently buried, our state association being the only mourners. Its members, who are not already members of the state society, will be very welcome there, and then the state society can again send delegates to the National body, which has finally adopted its views. It would be perfectly absurd to try to punish the New York State Medical Society by continuing the existence of the seceding body, when its action has been at last so thoroughly endorsed. We hope to see but one state society in New York next year.

### RECENT REVISION OF THE CODE OF ETHICS.

[*Medical Sentinel*, June, 1903.]

The report of the committee on ethics and its adoption, as modified by the house of delegates of the American Medical Association at New Orleans, marks a distinct step in advance. It carries out into practical ethics the principles, which have been foreshadowed now for three years past in the great movement looking towards the harmonisation and unification of the profession. It could hardly be otherwise; the spirit of revision is in the air, it has already reached the Scriptures, and has extended to that even more impregnable rock of doctrine, the Presbyterian Creed. Unless we wish to be classed with, or even behind theologians, we must submit to the same process. The keynote of the new code is squarely struck in Chapter III., in the statement that the local societies should consider as eligible to membership "any physician recognised as such by law, and possessing a good personal and professional character, whatever may be his individual views on any questions connected with the science of medicine." This frankly and completely ignores all "pathies," whether socalled "allopaths, homeopaths or hydropaths," and is, in our judgment, the only tenable ultimate position. The sooner local societies come into line with this declaration, the better it will be for the profession, as well as for them· personally. There was a time when the physician had practically no protection from either Church or State; when his only means of determining his standards and of maintaining the dignity of the profession lay in his own private organisation or school. Naturally these schools came to be mutually exclusive and intolerant, but the day, happily, has passed; in every civilised community the state now protects the health of its citizens to the extent of declaring that no one shall be permitted to bear the proud title of doctor and practise medicine, who cannot show to a competent examining body that he possesses adequate qualifications for this great responsibility.

When the state has declared a man a physician and fit to be trusted with the responsibility of human life, it is no longer necessary that this judgment should be confirmed by any school of medicine, whether it be the one to which we belong, or any other. That man is a physician in the eye of the law, and responsible to the law for any incompetency or immorality. He is also a physician in the eye of the public, and the old Anglo-Saxon principle that every man shall be deemed innocent until he shall be proven guilty, demands that he should be recognised as such, until by his life and conduct he has shown himself to be unworthy of confidence. The moment that he has done so, then our remedy is clear and should be exercised at once. We should demand the revocation of his certificate by the State Board, and expel him from our societies. With a society based upon this broad ground, expulsion from its ranks would practically mean expulsion from the profession, and would be treated accordingly.

### NEW ATTITUDE TOWARD CONSULTATIONS.

This, of course, cuts the Gordian knot of the consultation problem, which has been at the bottom of all the serious divisions in the profession for the last 25 years, notably in the State of New York. And it is time that it was so cut, for in our former attitude upon this subject, while jealously anxious for the standard and honor of our profession, we have been, to my eye, both negligent and almost criminally indifferent to a far more important element, and that is the welfare of the patient. Our former attitude practically amounted to this, that because a man chose to employ as his attendant a member of the profession with whose views upon certain therapeutical or pathological questions we were not entirely in harmony, therefore he and his family were to be utterly deprived of our skill and our knowledge, when demanded in consultation, even in the face of death. Of course we had a socalled emergency clause, according to which, to save life, we might soil our precious fingers by contact with an irregular, but this was of little value, and the general public, in my judgment, was perfectly right in regarding our attitude in this respect as both unreasonable and little short of inhuman. The patient has some rights, which even the warring schools of his physicians are bound to respect. It is a good thing that this barrier is down, also for another reason, and that is that while a considerable proportion of our profession unquestionably strictly adhered to its requirements, and thereby frequently deprived innocent women and children of their valued services in critical situations, yet a large minority of the profession, among them many of those who were most vociferous in denouncing the possibility of the admission of the socalled irregulars to membership in our societies, in the intervals between these paroxysms of protest, consulted with them freely and indiscriminately whenever the fee was large enough or the family sufficiently influential. The new departure will not merely form a broader and better protection for the interests of the patient and the best interest of the community, but will also relieve a minority of our members from the stain of insincerity and the painful necessity of hypocrisy. We have now a code so simple that its obligations are capable of prompt recognition by any man whose heart is in the right place, inside or outside of the profession, which is more than could be said before.

